# Variations in Fungal Community and Diversity in Doushen With Different Flavors

**DOI:** 10.3389/fmicb.2020.00447

**Published:** 2020-03-20

**Authors:** Qiaoqiao Luo, Yan Zhu, Zhongming Zhang, Yingying Cao, Weibing Zhang

**Affiliations:** College of Food Science and Engineering, Gansu Agricultural University, Lanzhou, China

**Keywords:** Doushen, high-throughput sequencing, flavor, fungal community, diversity

## Abstract

Doushen, a traditional Chinese fermented soybean product, may be spiced or spicy depending on whether pepper powder is added. While numerous studies have investigated the microbial communities of other fermented foods, little is known on the fungal diversity of Doushen. Therefore, in this study, we investigated the fungal community and diversity in both spiced and spicy Doushen. Our results revealed that fungal species richness significantly differed between the samples with different flavors. A total of nine phyla and 188 fungal genera were identified, and Ascomycota and *Aspergillus* were predominant in all samples. Based on linear discriminant analysis, a total of 57 OTUs were significantly different between the two samples. Results of non-metric multidimensional scaling and unweighted pair-group analysis suggested that the presence of pepper powder affects the microbial community in Doushen. Network analysis showed that microbial interactions between fungal communities in Doushen with different flavors were significantly different. The results on the enumeration and identification of fungi were consistent with the composition of the dominant genera in the samples with different flavors. This study provides a theoretical basis for future research on food ecology in Doushen.

## Introduction

**FIGURE 1:**
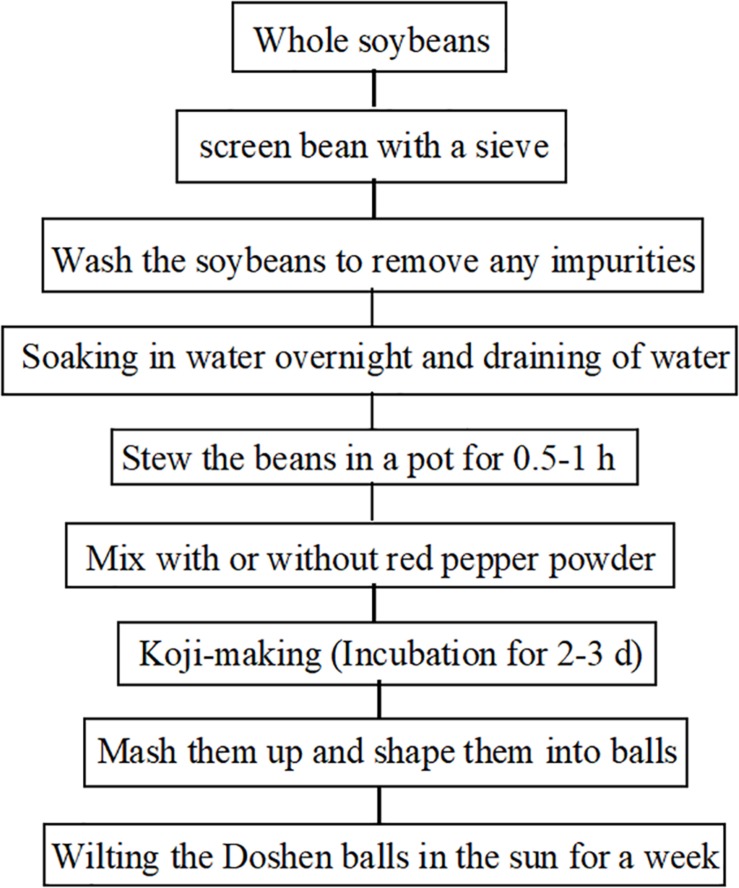
A schematic diagram of the production of Doushen.

Traditional fermented soybean-based foods, including natto (Liu et al., 2018), tempeh (Noriyuki, 1990), douchi (Zhang et al., 2018), sufu (Qiu et al., 2018), miso (Jayachandran and Xu, 2019), soy sauce (Li et al., 2018), and soybean paste (Inoue et al., 2016) have been consumed in Asia for centuries. Currently, these foods are becoming recognized and accepted by Western countries (Wang et al., 2007; Zhang et al., 2007; Wang et al., 2010). Doushen, a douchi-like product, has been produced and consumed widely as seasoning in Henan Province for thousands of years. Due to its delicious taste and unique flavor, Doushen is being favored by local people in Zhoukou, Xuchang, Shangqiu, and Kaifeng. The traditional preparation process of Doushen consists of screening, cleaning, soaking, cooking, mixing, koji-making, mashing, and wilting in the sun (Figure 1). The end products are processed into Doushen balls of approximately 4–6 cm in diameter. During the production of Doushen, raw soybeans are heat-treated sufficiently to kill most of the microorganisms except for the heat-resistant ones. However, cooked soybeans are fermented under exposed conditions; hence, a variety of endogenous and exogenous microorganisms may be involved in the fermentation process. After fermentation, soybeans used in Doushen are usually ground and sun-dried. Environmental factors might shape the microbial communities in Doushen. Therefore, abundant and diverse microbial populations may exist in this specific food ecosystem.

A variety of fungi, including *Aspergillus*, *Mucor*, *Rhizopus*, *Saccharomyces*, *Pichia*, and *Lichtheimia*, play a pinnacle role in the production of traditional fermented soybean products (Chen et al., 2011; Yang et al., 2016; Zhang et al., 2018). During the process of fermentation, fungi produce various enzymes including glutaminase, cellulase, proteases, lipases, amylases, and fibrinolytic enzymes. Under the action of these enzymes, proteins, sugars, lipids, and other compounds in soybeans are degraded into ketones, esters, and acids, thereby contributing to taste and aroma (Kim et al., 2010; Yebra and Monedero, 2010; Chen et al., 2012; Chen et al., 2014). Over the last four decades, several studies have analyzed the fungal community of traditional fermented soybean products using culture-dependent or culture-independent methods (Kim et al., 2010; Chen et al., 2011; Chen et al., 2012; Nam et al., 2012; Chen et al., 2014; Ji et al., 2014; Yang et al., 2016; Zhang et al., 2018). To date however, no studies have evaluated the microbial community in Doushen.

Traditional culture-dependent and culture-independent approaches have been used to investigate the microbial community of fermented soybean products. These methods provide little information on microbial community composition and structure (Kim et al., 2010; Chen et al., 2011; Chen et al., 2012, 2014). Recently, high-throughput sequencing has been used in ecosystem exploration. This technology circumvents the limitations associated with culture-dependent methods and, consequently, has been widely applied to analyze the microbial diversity in various natural environments such as soil, air, water, plants, and various human microbiota (Arumugam et al., 2011; Fu et al., 2015; Helena et al., 2016; Qin et al., 2016; Kozyaeva et al., 2017). Recently, numerous traditional fermented foods such as douchi (Yang et al., 2016; Zhang et al., 2018), soybean paste (Nam et al., 2012), cheese (Ahram et al., 2018), yogurt (Lisa et al., 2013), Qula (Zhu et al., 2018), and Pu-er tea (Zhang et al., 2016) have been analyzed using high-throughput sequencing In this study, we used high-throughput sequencing to evaluate the fungal community diversity in Doushen with different flavors obtained from Henan province. Our findings may assist in understanding the complex fungal community structure in Doushen, thereby providing a solid theoretical basis to select appropriate strains suitable for high-quality Doushen.

## Methods

### Sample Collection

Homemade Doushen samples with different flavor (spicy and spiced) were obtained from local producers in Fugou County (Henan province, China). Five Doushen balls of each flavor were randomly collected from one producer. A total of 30 samples were placed into an ice box for transportation to the lab. Each Doushen ball was divided into exterior and interior regions at a ∼2.5-cm depth from the surface using a saw. Two grams of each exterior or interior sample were collected from one ball. Subsequently, the five samples obtained from the same producer were pooled into a composite sample, and stored at −80°C. All the samples were divided into four groups based on sampling location and flavor. The first group includes the sample from the exterior of spicy Doushen (XLo), the second group includes the sample from the interior of spicy Doushen (XLi), the third group includes the sample from the exterior of spiced Doushen (WXo), and the fourth group includes the sample from the interior of spiced Doushen (WXi) (Table 1).

**TABLE 1:** Information of Doushen samples with different flavors.

Sample ID	Flavor	Sampling area	Sample location	Origin
XLo1	Spicy	Exterior regions of Doushen	China:Henan fugou	34.07 N 114.38 E
XLo2	Spicy	Exterior regions of Doushen	China:Henan fugou	34.07 N 114.38 E
XLo3	Spicy	Exterior regions of Doushen	China:Henan fugou	34.07 N 114.38 E
XLi1	Spicy	Interior regions of Doushen	China:Henan fugou	34.07 N 114.38 E
XLi2	Spicy	Interior regions of Doushen	China:Henan fugou	34.07 N 114.38 E
XLi3	Spicy	Interior regions of Doushen	China:Henan fugou	34.07 N 114.38 E
WXo1	Spiced	Exterior regions of Doushen	China:Henan fugou	34.07 N 114.38 E
WXo2	Spiced	Exterior regions of Doushen	China:Henan fugou	34.07 N 114.38 E
WXo3	Spiced	Exterior regions of Doushen	China:Henan fugou	34.07 N 114.38 E
WXi1	Spiced	Interior regions of Doushen	China:Henan fugou	34.07 N 114.38 E
WXi2	Spiced	Interior regions of Doushen	China:Henan fugou	34.07 N 114.38 E
WXi3	Spiced	Interior regions of Doushen	China:Henan fugou	34.07 N 114.38 E

### Physiochemical Characterization of Doushen

The moisture, lipid, and protein contents of samples were determined by AOAC methods (AOAC, 2000). Amino-type nitrogen and total acid contents were determined by the titration method described by Judoamidjojo (1986).

### Plate Count of Fungi

The total number of viable fungal cells in Doushen was determined using a standard viable cell counting method. Briefly, 1 g of Doushen samples was diluted to 100 mL normal saline (0.9% NaCl). The diluted samples were spread on potato-dextrose agar (PDA, BD, United States) for the enumeration of fungi. The agar plates were incubated at 28°C for 5-day, and the number of fungi in Doushen was calculated as colony forming units (CFU) per gram. The colonies on the plates were randomly selected and cultivated to extract total DNA. The internal transcribed spacer (ITS) region of nuclear ribosomal DNA was amplified using specific primers (ITS1 and ITS4). The PCR amplification products were purified and sequenced at Shanghai Personal Biotechnology Co., Ltd. (Shanghai, China). Sequences were blasted in NCBI web site for identification of fungi.

### DNA Extraction, PCR Amplification, and Sequencing

DNA of all Doushen samples was extracted with a Soil DNA Kit D5625-01 (Power Soil DNA Isolation kit, MOBIO Laboratories, Inc., United States) according to the manufacturer’s instructions. DNA concentration and quality was determined by using an ultraviolet visible spectrophotometer (Thermo Scientific, NC 2000, United States).

The universal primer pairs ITS1-F (CTTGGTCATTTAG AGGAAGTAA) and ITS2 (GCTGCGTTCTTCATCGA TGC) were used to amplify the fungal ITS rDNA gene targeting the ITS1-ITS2 region (Wu et al., 2018). The PCR thermal cycle profile was as follows: initial denaturation for 2 min at 98°C; followed by 25 cycles of denaturation at 98°C for 15 s, annealing at 55°C for 30 s, and extension at 72°C for 30 s; then a final extension at 72°C for 5 min, after which the samples were cooled to 10°C for later use.

All PCR products were sequenced on an Illumina Miseq PE300 Sequencing platform (Illumina, Inc., San Diego, CA, United States) at Shanghai Personal Biotechnology Co., Ltd. (Shanghai, China). Raw sequences were uploaded into the Sequence Read Archive at NCBI under the accession number PRJNA493155.

### Processing of High-Throughput Sequencing Data

Raw Illumina fastq files generated from the high-throughput sequencing were demultiplexed, quality filtered, and analyzed following pipelines of Mothur (V.1.31.2) and QIIME (V1.7.0) (Schlosset et al., 2009; Caporaso et al., 2010). Quality sequences were classified into operational taxonomic units (OTUs) with a cutoff of 97% identity using the *de novo* OTU selection strategy. Taxonomies were assigned by the RDP classifier (Version 2.2) (Cole et al., 2003) against the UNITE databases (Koljalg et al., 2013) with 80% confidence threshold.

### Data Analysis

Alpha diversity indices of Chao1, Shannon, and Good’s coverage were calculated using QIIME following the tutorial^1^ for each sample. The indices were subsequently tested for significant differences between the different groups using the Duncan’s test.

Non-metric multidimensional scaling (NMDS) and a Heat map were constructed using the R package^2^ (v2.15.3) to quantify differences in community composition. The difference of fungal community structures among Doushen samples was analyzed by Adonis permutational multivariate analysis (Adonis/PERMANOVA) and analysis of similarities (ANOSIM) (Mcardle and Anderson, 2001; Lozupone et al., 2007).

Unweighted pair-group method with arithmetic means (UPGMA) clustering was conducted using QIIME following the guidance^3^.

To identify differentially abundant taxa between different groups in Doushen samples, LDA effect size (LEfSe) was performed using the online Galaxy work flow framework^4^ (LDA score ≥2.0 and *p* ≤ 0.05; Segata et al., 2011).

The SparCC algorithm was used to analyze the correlation of all fungal genera in the abundance and variety of the Doushen samples, including positive correlation and negative correlation (Friedman and Alm, 2012). Statistical analysis was also performed on the screening of the correlation score >0.6 with a significance level less than 0.05. Then correlation networks were made using the biocloud tools, a free online platform for data analysis.^5^

## Results

### Physiochemical Characterization of Doushen

There was no significant difference in protein content between the spicy and spiced Doushen samples (*p* > 0.05; Table 2). However, there were differences in the amino-type nitrogen content (*p* < 0.05). In addition, no significant differences were obtained in fat and total acid content between the two types of Doushen samples (*p* < 0.05).

**TABLE 2:** Physiocheinical characterization of Doushen and enumeration of fungi.

Parameters	Spicy Doushen		Spiced Doushen	
	XLo	XLi	WXo	WXi
Fat (%)	1.63 ± 0.07^a^	1.58 ± 0.03^a^	1.47 ± 0.23^a,b^	1.42 ± 0.12^a,b^
Total acid (mg/g)	0.41 ± 0.01^a,b^	0.42 ± 0.01^a,b^	0.51 ± 0.00^a^	0.52 ± 0.01^a^
Amino-type nitrogen (%)	16.11 ± 0.03^a^	16.09 ± 0.03^a^	13.11 ± 0.03^b^	13.12 ± 0.02^b^
Protein (%)	21.93 ± 0.13^a^	21.88 ± 0.09^a^	21.76 ± 0.21^a,b^	21.79 ± 0.14^a,b^
Fungi (log CFU/g sample)	5.83 ± 0.09^a^	5.12 ± 0.02^b^	4.52 ± 0.07^c^	4.09 ± 0.05^d^

*Letters indicate Duncan’s pairwise differences among different samples (*p* < 0.05).*

### Sequencing and Classification

Total DNA was extracted from the Doushen samples, and the ITS rDNA gene was amplified by PCR. PCR products were sequenced using Illumina MiSeq platform at Shanghai Personal Biotechnology Co. Ltd. (Shanghai, China). After removing low quality and chimeric sequences, 774,761 high-quality sequencing reads were generated from 12 Doushen samples, with an average of 64,563 reads per sample (Table 3). A total of 9,051 OTUs were generated from the high-quality sequences, with an average of 754 OTUs per sample. OTUs were significantly higher in spicy Doushen samples (1,027 OTUs for XLo and 1,002 OTUs for XLi) than in spiced Doushen samples (615 OTUs for WXo and 373 OTUs for WXi). In addition, the samples from the exterior area (XLo and WXo) contained more OTUs than the samples from the interior area (Xli and WXi).

**TABLE 3:** Reads, OTUs, Good’s Coverage, Chaol, and Shannon’s indices for ITS rRNA sequencing of the Doushen samples.

Sample ID	Reads		OTUs		Good’s coverage		Chaol		Shannon	
	Mean	SD	Mean	SD	Mean (%)	SD (%)	Mean	SD	Mean	SD
XLo	74,974	8,043	1,027	111^a^	99.83	0.06	566	25^a,b^	5.72	0.08^a^
XLi	69,368	9,280	1,002	100^a,b^	99.83	0.12	403	13^c^	5.65	0.64^a^
WXo	65,726	8,386	615	91^c^	99.90	0.10	568	22^a^	5.60	0.14^a^
WXi	58,930	8,000	373	32^d^	99.90	0.00	253	17 ^d^	5.25	0.17^a,b^

*Letters indicate Duncan’s pairwise differences among different samples (*p* < 0.05).*

**FIGURE 2:**
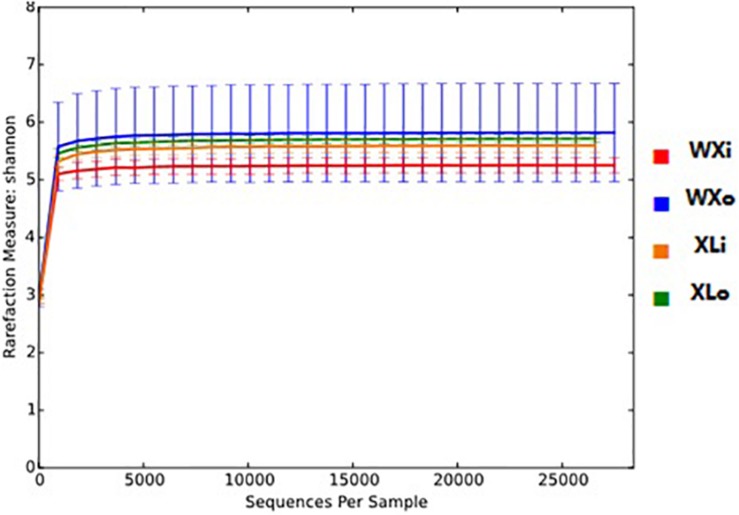
The rarefaction curve for Shannon diversity of different Doushen samples.

In the study, the Good’s coverage of all the samples was >98% (Table 3), indicating that the sequencing depth was sufficient to saturate the fungal diversity. The rarefaction curve for Shannon diversity indices was nearly parallel with the *x*-axis (Figure 2), suggesting that the majority of fungi present in Doushen had been captured.

### Analysis of Alpha Diversity

Shannon and Chao1 indexes were used to assess alpha diversity (Table 3). There were no significant differences in Shannon indexes among the samples (*p* > 0.05), implying that the fungal diversity difference of the Doushen samples was not significant. However, the results showed that fungal species richness was different (*p* < 0.05) between Doushen samples with different flavor. In addition, the results showed that the exterior area of Doushen samples had higher fungal diversity than the interior area, consistent with the differences in fungal counts and OTUs.

### Comparison of Fungal Communities

**FIGURE 3:**
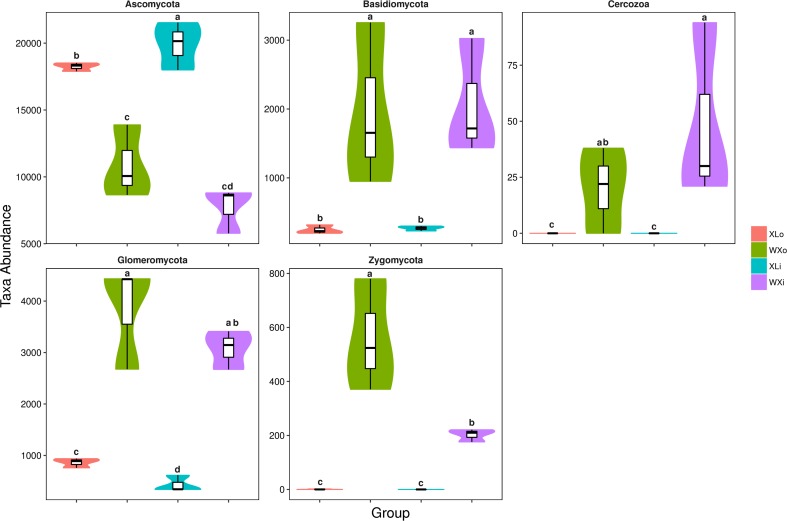
Taxonomic profiles of statistically significant fungi at the phylum level. Different letters indicate significance at *p* < 0.05.

To investigate the microbial community and diversity in Doushen, the fungal ITS rRNA gene sequences were classified at both phylum and genus levels. At the phylum level, there were nine phyla detected in Doushen samples. Ascomycota were predominant in WXi, WXo, Xli, and XLo, with a relative abundance >20% (Table 4). The relative abundance of Ascomycota in spicy Doushen was particularly high (>90%); its abundance was significantly different among XLo, WXo, and XLi (*p* < 0.05; Figure 3). Glomeromycota and Basidiomycota, which were detected in all Doushen samples, were particularly more abundant in spiced Doushen (*p* < 0.05; Figure 3). The abundance of Zygomycota was higher in spiced Doushen than in spicy Doushen (*p* < 0.05; Figure 3).

**TABLE 4:** Percentage of the main fungal phylum and genera in samples.

Fungi	Percentage composition in samples			
	XLo	XLi	WXo	WXJ
	*n* = 3	*n* = 3	*n* = 3	*n* = 3
**Phylum**				
Ascomycota	93.85	96.28	46.83	29.20
Glomeromycota	4.43	2.09	16.57	11.62
Basidioraycota	1.26	1.29	8.41	7.78
Zygomycota	0.00	0.00	2.41	0.76
Rozellomycota	0.00	0.00	0.33	0.74
Chytridiomycota	0.00	0.00	0.41	0.27
Cercozoa	0.00	0.00	0.09	0.18
Ciliophora	0.00	0.00	0.06	0.02
Neocal1imastigomycota	0.00	0.00	0.04	0.00
**Genus**				
*Aspergillas*	4.31	6.63	9.42	7.75
*Colletotiichtim*	15.76	12.93	0.26	0.08
*Candida*	0.02	0.01	8.76	0.29
*GibelJtdopsis*	0.76	0.47	2.00	4.94
*Diaporthe*	4.20	4.44	0.24	0.38
*Debaiyomyces*	0.00	0.00	6.70	0.59
*Simpticitthan*	0.00	0.03	1.53	3.51
*Bullera*	0.66	0.53	2.20	1.58
*Penicillhim*	0.04	0.03	2.08	0.74
*Aiix’tixaiiwit*	1.12	1.04	0.27	0.23
*Ciyptococcus*	0.05	0.12	1.67	0.43
*Thermoasciss*	0.00	0.01	1.57	0.38
*Malassezia*	0.00	0.00	1.04	0.89
*Wallemia*	0.14	0.23	0.37	1.18
*Cystobassdium*	0.00	0.00	0.01	1.58
*Rhodotanrfa*	0.00	0.00	0.27	0.61
*Gibberella*	0.37	0.57	0.10	0.00
*Tiicknsporon*	0.17	0.00	0.24	0.44
*Guehomyces*	0.03	0.03	0.63	0.08
*Fichia*	0.04	0.05	0.29	0.14

At the genus level, there were 188 genera tested in the Doushen samples. The relative abundance of the top 20 fungal genera is shown in Table 4. A total of 15 dominant genera were obtained in all samples, with a relative abundance >1%. *Aspergillus* was the only dominant genus present in all samples, which had a higher relative abundance in spiced Doushen (XLo and XLi). Three genera (*Colletotrichum, Diaporthe*, and *Acremonium*) were only dominant in spicy Doushen. Among them, *Colletotrichum* was the most-dominant genus, with a relative abundance >10%, followed by *Diaporthe* (>4%). Ten genera (*Candida*, *Gibellulopsis*, *Debaryomyces*, *Simplicillium*, *Bullera*, *Penicillium*, *Cryptococcus*, *Thermoascus*, *Malassezia*, *Wallemia*, and *Cystobasidium*) were only dominant in spiced Doushen. Among these, three dominant genera (*Gibellulopsis*, *Simplicillium*, and *Bullera*) were present in all spiced Doushen samples. Six genera (*Candida*, *Debaryomyces*, *Penicillium*, *Cryptococcus*, *Thermoascus*, and *Malassezia*) were only dominant in WXo samples (the exterior area of spiced Doushen). The other two genera (*Wallemia* and *Cystobasidium*) were only dominant in WXi samples (the interior area of spiced Doushen). Additionally, a total of 173 non-dominant genera (relative abundance <1%) were identified in the samples.

**FIGURE 4:**
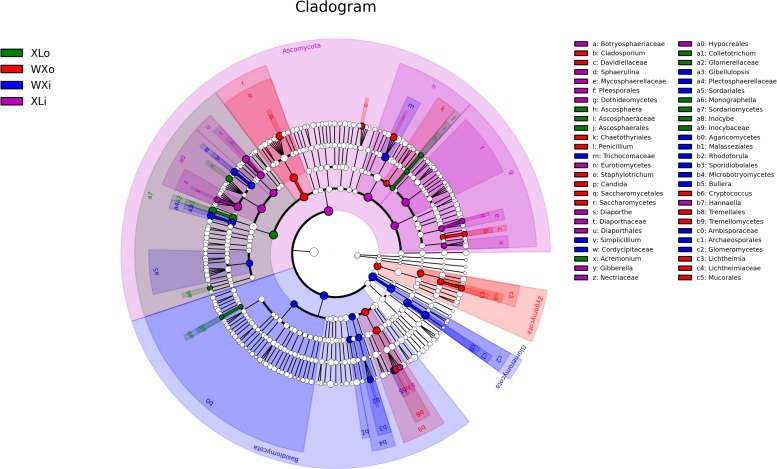
Linear discriminant analysis in microbial community compositions in Doushen samples. The node size represents the difference in relative abundance.

To identify the specific taxa within each group, LEfSe analysis was applied to compare the microbiota between different groups (Figure 4). In the XLi group, 14 taxa were enriched including one phylum (Ascomycota), two classes (Eurotiomycetes and Dothideomycetes), three orders (Diaporthales, Hypocreales, and Pleosporales), four families (Diaporthaceae, Botryosphaeriaceae, Mycosphaerellaceae, and Nectriaceae), and four genera (*Diaporthe*, *Hannaella*, *Gibberella*, and *Sphaerulina*). The fungal groups enriched in the XLo group were one class (Sordariomycetes), one order (Ascosphaerales), two families (Inocybaceae and Glomerellaceae), and five genera (*Ascosphaera*, *Inocybe*, *Colletotrichum*, *Acremonium*, and *Monographella*). In the WXi group, 15 taxa were enriched including one phylum (Zygomycota), two classes (Tremellomycetes and Saccharomycetes), four orders (Chaetothyriales, Mucorales, Saccharomycetales, and Tremellales), two families (Davidiellaceae and Lichtheimiaceae), and six genera (*Cryptococcus*, *Penicillium*, *Cladosporium*, *Staphylotrichum*, *Candida*, and *Lichtheimia*). The fungal groups enriched in the WXo group were two phyla (Basidiomycota and Glomeromycota), three classes (Microbotryomycetes, Glomeromycetes, and Agaricomycetes), five orders (Archaeosporales, Sporidiobolales, Trichocomaceae, Malasseziales, and Sordariales), three families (Plectosphaerellaceae, Cordycipitaceae, and Ambisporaceae), and four genera (*Gibellulopsis*, *Simplicillium*, *Rhodotorula*, and *Bullera*). In total, 57 OTUs were significantly different between samples with different flavors.

### Analysis of Beta Diversity

**FIGURE 5:**
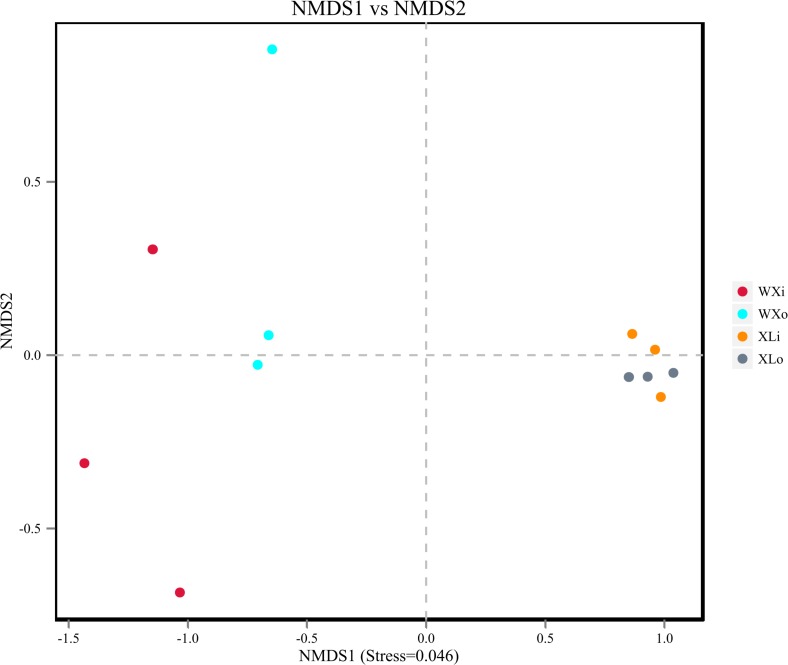
The beta diversity results of NMDS indicating the data distribution of doushen samples.

**FIGURE 6:**
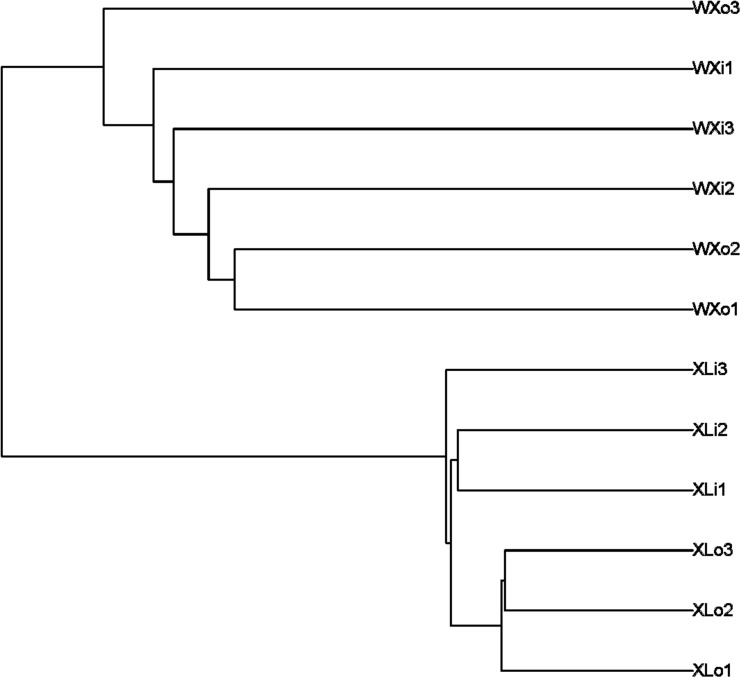
The unweighted pair-group analysis (UPGMA) based on UniFrac distance for fungal communities of Doushen samples.

Beta diversity results of NMDS based on unweighted uniFrac indicated the data distribution of Doushen samples and is shown in Figure 5. Samples of the same flavor grouped together tightly indicating that the fungal community structure of XLo and XLi has a high degree of similarity and small differences. This situation also applies to the fungal community structure of the WXo and WXi. In addition, the XL samples are more tightly clustered than WX samples in the present study. The results were much similar when comparing the samples with different flavors by using unweighted pair-group analysis (Figure 6). Adonis/PERMANOVA analysis was performed out on the sample, the results showed that *p* = 0.001 (*p* < 0.05). Results of ANOSIM’s statistical analysis also indicated that flavor (the addition of pepper powder) was a crucial factor influencing the fungal composition of the different samples (*R* = 0.6235, *P* = 0.002).

### Interactions of Fungi in Doushen Samples

**FIGURE 7:**
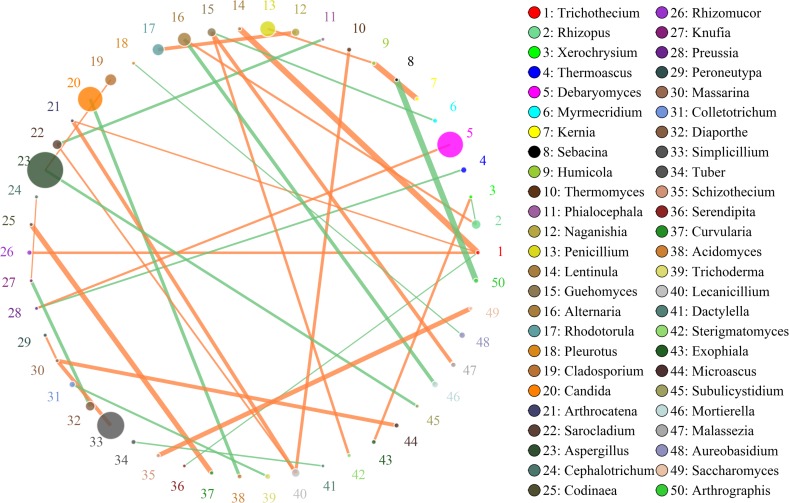
Networks of microbial interaction in the spicy doushen samples. Circle is on behalf of the species, size of the circle represents the abundance. Lines represent the correlation between the two species, line thickness to represent the strength of the correlation, the color of the line: orange represents the positive correlation, green represents the negative correlation.

**FIGURE 8:**
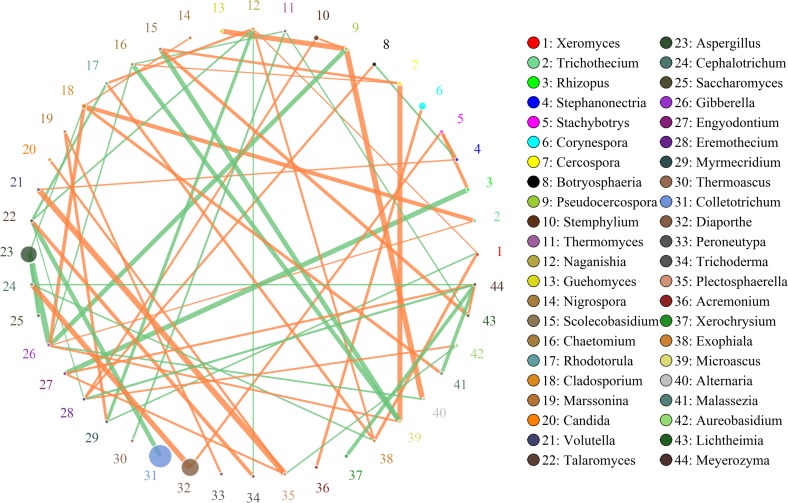
Networks of microbial interaction in the spiced doushen samples. Circle is on behalf of the species, size of the circle represents the abundance. Lines represent the correlation between the two species, line thickness to represent the strength of the correlation, the color of the line: orange represents the positive correlation, green represents the negative correlation.

In this study, SparCC algorithm was used to calculate the relationship between genera identified in Doushen and it was visualized as the network (v 3.7.1) (Figures 7, 8). The network for fungal communities in spicy Doushen consisted of 50 nodes and 34 edges (Figure 7). Results suggest the network is cooperative, and the ratio of cooperative and non-cooperative interaction is 20:14. *Trichothecium* had a negative relationship with *Serendipita*, but had a positive relationship with three other genera: *Lentinula*, *Arthrocatena*, and *Rhizomucor*. All effects related to *Massarina* were positive, including three other genera: *Peroneutypa*, *Microascus*, and *Slimplicillium*.

The network for microbial community in spiced Doushen consists of 44 nodes and 61 edges (Figure 8). There were three fungal genera were found to be hub genera (≥6 edges per node) in the network, including *Naganishia*, *Gibberella*, and *Meyerozyma*. Results suggest the network is cooperative, and the ratio of cooperative and non-cooperative interaction is 35:26. *Aspergillus* had a negative relationship with three other genera: *Cladosporium*, *Saccharomyces*, and *Gibberella*. All of the correlation coefficients associated with *Marssonina* were positively correlated. These included *Myrmecridium* and *Peroneutypa*. *Gibberella* was found to have a negative relationship with three genera: *Alternaria*, *Chaetomium*, and *Pseudocercospora*, while have a positive relationship with three other genera: *Cladosporium*, *Microascus*, and *Trichothecium*. *Cladosporium* had a positive relationship with three other genera: *Malassezia*, *Nigrospora*, and *Trichothecium*.

### Enumeration and Identification of Fungi

The number of fungal colonies in the WXo and WXi groups was 4.52 log CFU/g sample and 4.09 log CFU/g sample, respectively. The fungal counts in spicy Doushen were 5.83 log CFU/g sample in XLo and 5.12 log CFU/g sample in XLi. The enumeration results showed that the fungal population was lower in spiced Doushen than in spicy Doushen (*p* < 0.05). In addition, the results revealed that the number of fungi was lower in the interior area than in the exterior area of Doushen balls (Table 2).

**FIGURE 9:**
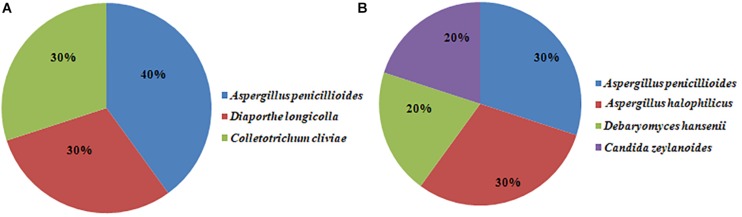
Fungal strains composition in spicy **(A)** and spiced **(B)** Doushen samples.

Twenty fungal strains were randomly selected from the Doushen samples and identified by ITS rRNA sequencing. *Aspergillus penicillioides*, *Diaporthe longicolla*, and *Colletotrichum cliviae* were detected in spicy Doushen and accounted for 40, 30, and 30%, respectively (Figure 9 and Table 5). *A. halophilicus*, *A. penicillioides*, *Debaryomyces hansenii*, and *Candida zeylanoides* were identified in spiced Doushen, accounting for 30, 30, 20, and 20%, respectively (Figure 9 and Table 5). These results were consistent with the composition of the dominant genera in the samples with different flavors.

**TABLE 5:** The closest related type strains of sequences of isolates from Doushen in NCBI Genbank.

Isolates	Closest relative sequence	Accession number	Similarity (%)	Origin
D1	*Aspergillus penicillioides* NRRL 4548	AB006429.1	99	Spicy/spiced Doushen
D2	*Diaporthe longicolla* strain D25.1	MK483177.1	99	Spicy Doushen
DSB3	*Colletotrichum cliviae* strain *JQS*	KX957763.1	98.1	Spicy Doushen
DSB4	*Aspergillus halophilicus* isolate NRRL2739	EF652088.1	98	Spiced Doushen
DSB5	Debaryomyces hansenii strain CBS 767	MK394104.1	99	Spiced Doushen
DSB6	Candida zeylanoides culture CBS:4909	KY106917.1	99	Spiced Doushen

## Discussion

Different types of traditional fermented soybean products with special flavor have been produced and consumed around the world for centuries (Noriyuki, 1990; Wang et al., 2010; Li et al., 2018; Liu et al., 2018; Qiu et al., 2018; Zhang et al., 2018). Doushen, a douchi-like fermented soybean product, has been produced in Henan Province for a long time. The differences between Doushen and other traditional fermented soybean products is that the soybeans used for Doushen need to be minced. The study of microbial mechanisms is gaining considerable attention within the scientific community. In this study, the fungal diversity in two types of homemade traditional Doushen was evaluated by high-throughput sequencing. A total of nine fungal phyla and 188 fungal genera were detected in the Doushen samples. At the phylum level, Ascomycota, Basidiomycota, Rozellomycota, Chytridiomycota, Glomeromycota, and Zygomycota were previously detected in Douchi. However, Cercozoa and Ciliophora have not been previously detected in Douchi (Chen et al., 2011; Zhang et al., 2018). At the genus level, *Aspergillus*, *Diaporthe*, *Colletotrichum*, *Gibellulopsis*, *Simplicillium*, *Bullera*, *Penicillium*, *Acremonium*, *Cryptococcus*, *Wallemia*, *Pichia*, *Yarrowia*, and *Candida* were also detected in Douchi (Chen et al., 2011; Ji et al., 2014; Zhang et al., 2018). Among them, the relative abundance of *Pichia*, *Candida*, and *Yarrowia* was comparatively lower (Zhang et al., 2018). *Aspergillus*, *Diaporthe*, *Colletotrichum*, *Gibellulopsis*, and *Simplicillium* have a higher relative abundance in Doushen than in douchi (Zhang et al., 2018). However, *Debaryomyces* and *Cystobasidium* were not detected in a previous study (Zhang et al., 2018). The findings revealed a plethora of different types of fungi in the samples. Fermented soybean products are usually prepared with cooked soybeans and contain both endogenous and exogenous microorganisms involved in the fermentation process. Therefore, there is a variety of microorganisms present in the fermented soybean products (Chen et al., 2011; Yang et al., 2016).

Our study also demonstrated that the microorganisms in Doushen samples with different flavors exhibit a large degree of biodiversity. At the phylum level, the relative abundance of Basidiomycota, Cercozoa, and Zygomycotawas significantly different between spicy Doushen and spiced Doushen (Figure 3 and Table 4). The same applies to the relative abundance of *Colletotrichum* and *Candida* at the genus level (Table 4). The present study also showed that specific members of the fungal genera were dominant in the Doushen samples with different flavors. *Aspergillus*, *Colletotrichum*, *Diaporthe*, and *Acremonium* were the predominant fungi in spicy Doushen, whereas *Aspergillus*, *Candida*, *Debaryomyces*, *Gibellulopsis*, *Simplicillium*, *Bullera*, *Penicillium*, *Cryptococcus*, *Thermoascus*, and *Malassezia* were predominant in spiced Doushen. Given that Doushen is naturally fermented from steamed soybeans and do not require a starter, these fungi may emerge from the environment or other raw materials. There have been some reports that soybeans can be the main source of microorganisms in soybean fermentation (Mulyowidarso et al., 1989; Mulyowidarso et al., 1990; Ji et al., 2014). The present study showed that fungal diversity in different parts of Doushen samples was significantly different. These results imply that aeration and water content may be another important determinant, as previously reported in doenjang-meju (Ji et al., 2014). Chili Pepper, a key ingredient of several fermented soybean products, is present in Doushen products. Doushen with different flavors (spicy and spiced) were prepared with and without red pepper powder. Previous studies have revealed that chili pepper affects the microbial distribution in Kimchi and intestinal flora (Jeong et al., 2013; Cao et al., 2019). In this study, besides the significant difference in fungal composition between two types of Doushen samples, we found that the XL samples were more tightly clustered than WX samples (Figure 5). The result indicated that pepper powder exerted a very strong selection on fungal community structure of Doushen samples. Due to the absence of pepper powder, the fungal communities in the WX samples were more random than that in the XL samples. Previous studies have indicated that both deterministic and stochastic processes play an important role in microbial community assembly in natural environments (Chen et al., 2019; Hou et al., 2019; Gao et al., 2020). Future studies should use the ecological null models to decipher the relative importance of deterministic and stochastic processes in the microbial communities of Doushen.

Additionally, we obtained significant differences in microbial interactions between Doushen samples with different flavors, indicating that the Doushen microbial ecosystem may depend on different interactive relationships. Unique co-occurrence patterns were observed for Doushen samples with different flavors, which is likely to reflect adaptation to different environmental and nutritional conditions. Different types of fungi have different roles in soybean fermentation and could result in a complex relationship between species diversity and ecosystem function. *Aspergillus*, the most common genus of mold found in the environment, includes approximately 185 species, 20 species of which are harmful to humans and animals (Moy et al., 2012). However, *Aspergillus* was the predominant fungus in Douchi during koji-making and has been detected in meju, soy sauce, sofu, bean paste, and other traditional fermented soybean products (Lee et al., 2010; Moy et al., 2012; Li et al., 2017; He et al., 2019). Within the *Aspergillus* genus, the most common species is *A.oryzae*, which secretes enzymes that digest raw materials during soybean fermentation (He et al., 2019). *Candida*, a genus of yeast-like fungi, comprises pathogenic species that cause oral thrush. However, some species of *Candida* play important roles in enhancing the taste and quality of fermented soybean products, such as Douchi and soy sauce (Suezawa et al., 2006; Cao et al., 2010; Ying et al., 2010; Feng et al., 2012). *Debaryomyces* is an ascomycetous yeast species present in traditional fermented soybean foods such as Sufu, soybean pastes, doenjang, and kanjang (Azizul et al., 2015; Sun et al., 2018). The best-known species of this genus, *D. hansenii*, can grow under high salt concentrations and can secret various peptidases and proteases, which contribute to the aroma and flavor of fermented foods (Azizul et al., 2015). *Cryptococcus* is a genus of encapsulated yeast, which is found in fermented cereals, traditional llama meat sausages, traditional fermented soybean foods, alcoholic beverages, cheese, fermented pepper, and soy sauce (Corsetti et al., 2001; Volkmar et al., 2009; Lucía et al., 2014; Azizul et al., 2015; Zhao et al., 2016). Several species of *Cryptococcus* can produce extracellular enzymes such as lipase, pectinase, cellulase, xylanase, and laccase (Gomes et al., 2000; Kamini et al., 2000; Nahoko et al., 2012). The genus *Thermoascus* includes some species that can grow at high temperatures (Hosoya et al., 2014). These fungi have been identified in tea, olive cake, fruit juice, and other agricultural products and can produce amylase, xylanase, and cellulase (Adams, 1992; Wareing, 1997; Ping et al., 2018). *Bullera*, a genus belonging to *Ballistosporous*, can produce conidia and are present in diverse plants (phyllosphere), dairy products, and various environments (Nakase, 2000; Mushtaq et al., 2013; Silva-Bedoya et al., 2014). Some species of *Bullera* are producers of β-galactosidase, toxins, and coenzyme Q_10_ (Golubev and Nakase, 1998; Cho et al., 2003). Other genera, such as *Gibellulopsis*, *Simplicillium*, *Penicillium*, *Colletotrichum*, and *Diaporthe*, mainly include a number of fungal pathogens (Araceli et al., 2010; Udayanga et al., 2012). Numerous species of these genera produce extracellular enzymes such as glucoamylase, lipase, pectinase, and cellulase, which may affect product quality (Bahkali, 1992; Araceli et al., 2010; Prajapati et al., 2013). Some species of *Penicillium*, *Colletotrichum*, and *Diaporthe* produce fungal toxins under certain conditions (Kung et al., 1976; Gunnar et al., 2012; Zhang et al., 2012). In the present study, the exact function of the microorganisms has not been determined for lack of information on the species. Therefore, to assess their role on Doushen quality, future studies should isolate and grow these fungi.

## Conclusion

High-throughput sequencing is a powerful method for exploring a large diversity of microorganisms in an array of environments. Until recently, few studies have focused on food microbiota. In this study, fungal diversity and communities in Doushen samples with different flavors were studied using high-throughput sequencing. This is the first study that applies this technology to study food ecology in Doushen. The results provided detailed information on the fungal diversity of Doushen, which is important for the production of Doushen.

## Data Availability Statement

The datasets generated for this study can be found in the PRJNA493155.

## Author Contributions

QL, ZZ, and WZ contributed conception and design of the study. YZ is co-first author. ZZ and YZ performed the statistical analysis. QL and YC wrote the first draft of the manuscript. All authors contributed to manuscript revision, read and approved the submitted version.

## Conflict of Interest

The authors declare that the research was conducted in the absence of any commercial or financial relationships that could be construed as a potential conflict of interest.

## Footnotes

1 http://qiime.org/scripts/alpha_diversity.html

2 https://www.r-project.org/

3 http://qiime.org/1.4.0/scripts/jackknifed_beta_diversity.html

4 http://huttenhower.sph.harvard.edu/galaxy/

5 https://international.biocloud.net/zh/software/tools/
